# Nonuniform gene expression pattern detected along the longitudinal axis in the matured rice leaf

**DOI:** 10.1038/srep08015

**Published:** 2015-01-26

**Authors:** Ning Li, Yun-Ru Chen, Zehong Ding, Pinghua Li, Ying Wu, Ai Zhang, Sheng Yu, James J. Giovannoni, Zhangjun Fei, Wei Zhang, Jenny Z. Xiang, Chunming Xu, Bao Liu, Silin Zhong

**Affiliations:** 1 Key Laboratory of Molecular Epigenetics of the Ministry of Education, Northeast Normal University, Changchun 130024, China; 2 State Key Laboratory of Agrobiotechnology, the School of Life Sciences, The Chinese University of Hong Kong, Hong Kong, China; 3 The Institute of Tropical Bioscience and Biotechnology, Chinese Academy of Tropical Agricultural Sciences, Hainan, Haikou, P. R. China; 4 College of Agriculture, Shandong Agricultural University, Shandong, China; 5 Boyce Thompson Institute for Plant Research, Cornell University, Ithaca, NY, USA; 6 Weill Cornell Medical College, Cornell University, NY, USA

## Abstract

Rice (*Oryza sativa*) is a staple crop that supports half the world's population and an important monocot model system. Monocot leaf matures in a basipetal manner, and has a well-defined developmental gradient along the longitudinal axis. However, little is known about its transcriptional dynamics after leaf maturation. In this study, we have reconstructed a high spatial resolution transcriptome for the matured rice leaf by sectioning the leaf into seven 3-cm fragments. We have performed strand-specific Illumina sequencing to generate gene expression profiles for each fragment. We found that the matured leaf contains a longitudinal gene expression gradient, with 6.97% (2,603) of the expressed genes showing differentially expression along the seven sections. The leaf transcriptome showed a gradual transition from accumulating transcripts related to primary cell wall and basic cellular metabolism at the base to those involved in photosynthesis and energy production in the middle, and catabolic metabolism process toward the tip.

Monocot or cereal crops such as rice, maize, wheat and barley provide the majority of the calories in human diets, and their leaf tissues are the most important source of the photosynthetic energy. Unlike dicot, monocot has distinct leaf architecture with a basipetal developmental gradient (maturation from tip to base) along the leaf blade^1^. Understanding the transcriptional machinery within the leaf will provide crucial information for future crop improvement to meet the ever-increasing demand by the rapidly growing population.

It has been shown that a gene expression gradient exists in the developing rice and maize leaves and the transcriptome inside a developing leaf is highly dynamic^2^,^3^,^4^. The leaf sheath is responsible for primary cell wall and basic cellular metabolism, while the leaf blade tends to express more genes related to secondary cell wall biosynthesis and photosynthesis. However, most of the photosynthesis is carried out after leaf maturation, and little is known about whether a matured leaf would have a developmental gradient and whether the gradient observed in the maize is conserved in plants that operate C3 photosynthesis such as rice.

In this study, we examined the gene expression patterns within the matured rice leaf by dividing the fully expanded rice leaf blade it into seven 3 cm consecutive sections, and performed strand-specific RNA sequencing to generate a comprehensive transcriptome database for the matured rice leaf (http://137.189.43.55/JBrowse/). Our data revealed the existence of a dynamic gene expression gradient within the matured leaf and could provide a valuable genetic resource for C3 monocot research and future crop improvement programs.

## Results

### Transcriptome profiles of rice leaf longitudinal sections

**Figure 1:**
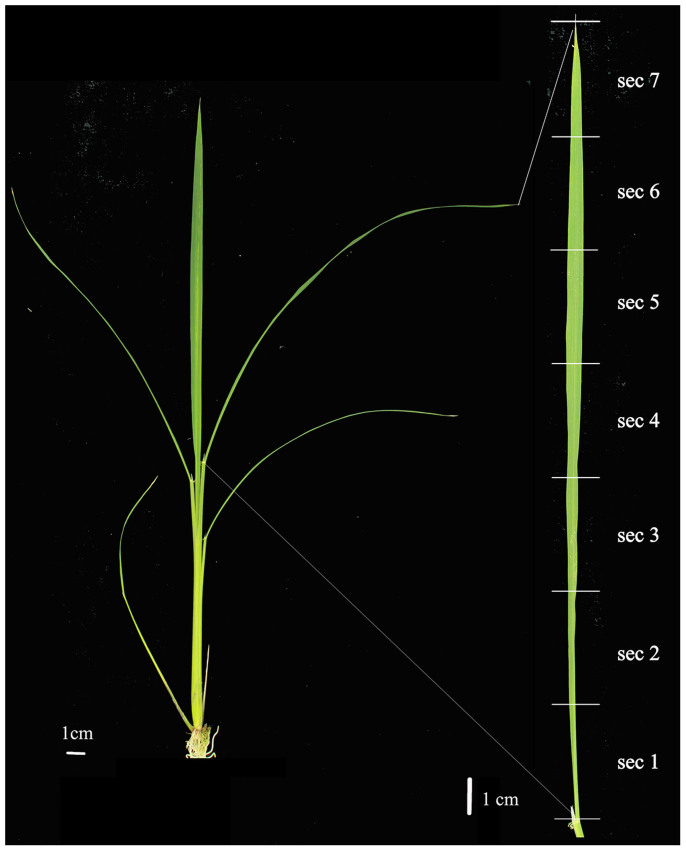
The matured rice leaf and the sections used for RNA-Seq analysis. (A) The rice seedling and (B) seven 3 cm sections from bottom to tip used for the experiment.

We constructed 14 strand-specific RNA-Seq libraries for seven 3-cm consecutive rice leaf sections with two biological replicates (Fig 1). These sections were referred to as section number 1 (leaf base) to 7 (leaf tip). After filtering out the adapter, low quality and ribosomal RNA reads, we obtained 48 million cleaned 51 nt single end reads (Table S1). Around 39 million cleaned reads (81%) could be aligned to the rice genome.

**Figure 2:**
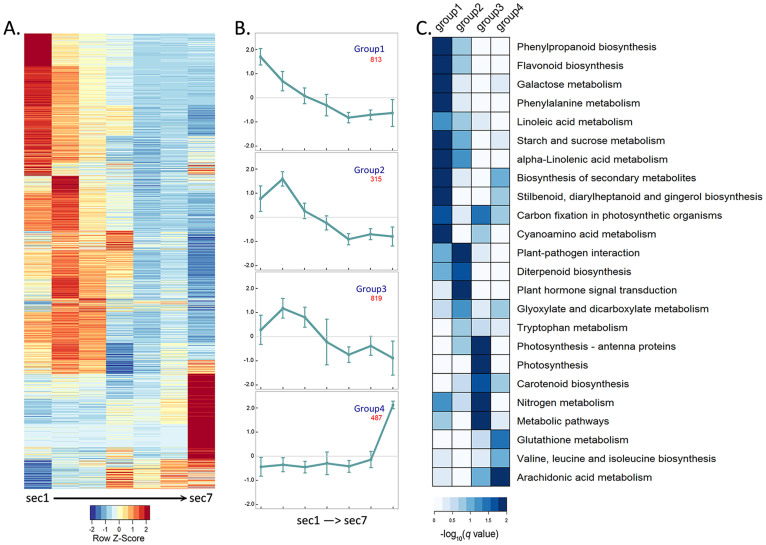
Gene expression pattern of seven leaf sections. (A) 2,603 differentially expressed genes have been identified in the seven 3 cm leaf sections. Heatmap showing the cluster of the DEG gene expression levels in each section (from section 1 to 7, leaf base to leaf tip). (B) The expression trends of DEGs in 4 main categories clustered by K-means algorithm, and the gene number contained by each group. (C) Heatmap of KEGG enrichment for the 4 categories (-log10(q value) of each pathway).

We detected the expression of 37,361 genes (67.33%) in at least one of the seven sections, and 18,992 genes (33.92%) in all seven sections. The numbers of expressed genes are similar in all sections, with 30,321, 29,649, 29,628, 28,986, 30,200, 29,552 and 30,340 expressed genes identified in sections 1 to 7, respectively. In total, 2,603 differentially expressed genes (DEGs) (6.97% of all expressed genes) were identified in at least one pairwise comparison between the sections, and 142 out of 2,603 DEGs have ORGO annotation (Fig. 2, Table S2). DEG analysis showed that the expression patterns of adjacent sections are more similar, and the expression divergences correlated well with the physical distance of the sections (Table S2–3). Our result also clearly demonstrated that the transcriptome within the matured rice leaf is not static, and the leaf contains a dynamic transcriptional gradient along the proximal-distal leaf axis.

### Functional category and cluster of DEGs

GO, AgriGO and KEGG enrichment analysis indicated that the 2,603 DEGs we identified are associated with various biological processes, molecular functions, cellular components and pathways (Table S3–7). We clustered the DEGs into four groups based on their expression pattern along the leaf sections. GO enrichment analysis were performed for each group to identify the common and different pathways in the particular categories representing functional divergence of the different leaf sections (Fig S2). The first group represents genes highly expressed near the base of leaf and contains genes involved in the primary and secondary metabolism such as enzymes in phenylpropanoid biosynthesis, starch and sucrose metabolism and flavonoid biosynthesis. The second group contains genes whose expression peak in the second leaf section. Pathways associated with cyan-amino acid metabolism, plant pathogen interaction, diterpenoid biosynthesis and hormone signaling were significantly enriched. The third group represents genes that are highly expressed in the section 2 and 3. It includes many genes related to photosynthesis such as those encoding antenna proteins, pigment biosynthesis and nitrogen metabolism. Genes in the forth group show highest expression in the leaf tip, and they are associated with the glutathione metabolism, valine, leucine and isoleucine biosynthesis, and arachidonic acid metabolism.

### Expression of photosynthesis-related genes in the middle leaf sections

Leaf blade is the major organ for photosynthesis, and photosystem differentiation is tightly linked to the leaf development. In term of sheer transcript abundance, the middle sector of leaf blade accumulated more photosynthesis related transcripts. Our KEGG enrichment analysis of DEGs revealed that many enriched pathways in the middle sector (cluster group 3) are related to photosynthesis (Fig 2C, Table S7). The expression trends of genes in these enriched pathways corresponded well with the results of the GO enrichment analysis, which showed most of genes involved in photosynthesis and energy biosynthesis are differentially expressed in group 3 (Fig S1B).

We further examined the expression pattern of several key genes involved in photosynthesis, such as the genes encoding the antenna proteins that are responsible for light-harvesting^5^. In the KEGG item of photosynthesis antenna protein, 13 out 15 genes are differentially expressed along the sections (Fig. S2A), and we have also performed qRT-PCR to confirm their RNA-Seq results (Table S8). The expression of these photosynthesis related genes first rise to the highest level in section 2, and starts to decrease in sections 3 and 4. Similarly, the ribulose 1,5-bisphosphate/oxygenase (RuBisCO) genes, whose expressions are well known to correlate with the net rate of photosynthesis and encode the key enzyme for carbon fixation, showed a similar expression pattern in the middle sectors^6^. In our study, 3 of 4 RuBisCO small subunit coding genes (LOC_Os12g17600, LOC_Os12g19381, and LOC_Os12g19470) were found to be highly expressed in the middle of leaf, suggesting the presence of a photosynthesis gradient similar to those observed in the maize leaf^2^ (Fig. S2A).

### Differential expression of transcription factors

**Figure 3:**
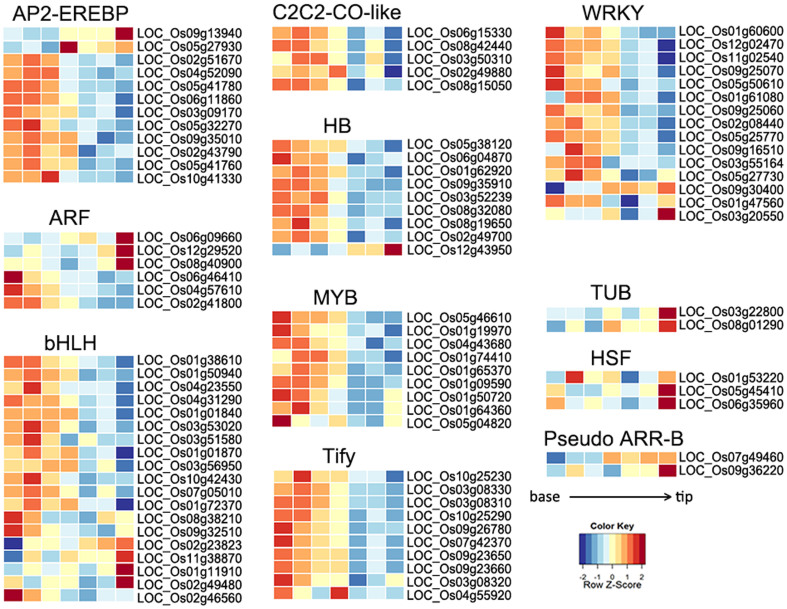
Expression pattern of the transcription factors in the seven leaf sections. The expression of 1,864 transcription factor genes could be detected in at least one leaf section, and 188 were differentially expressed along the leaf developmental gradient.

Our data also revealed a longitudinal dynamics of transcription factor gene expression. According to the annotation of PlantTFDB^7^,^8^, the expression of 1,864 transcription factor genes could be detected in our dataset, and 188 (10.09%, representing 43 families) were differentially expressed along the leaf developmental gradient (Fig 3 and S4; Table S9 and S10). Enrichment analysis indicated that five TF families are significantly enriched in the DEGs, and many of their members are highly expressed in the leaf base (Table S10 and S11). For example, members of WRKY, Tify, MYB, HB, bHLH, AP2-EREBP were highly expressed near the base, and only a few genes from TUB, HSF and Pseudo ARR-B TF families were highly expressed in leaf tip.

## Discussion

**Figure 4:**
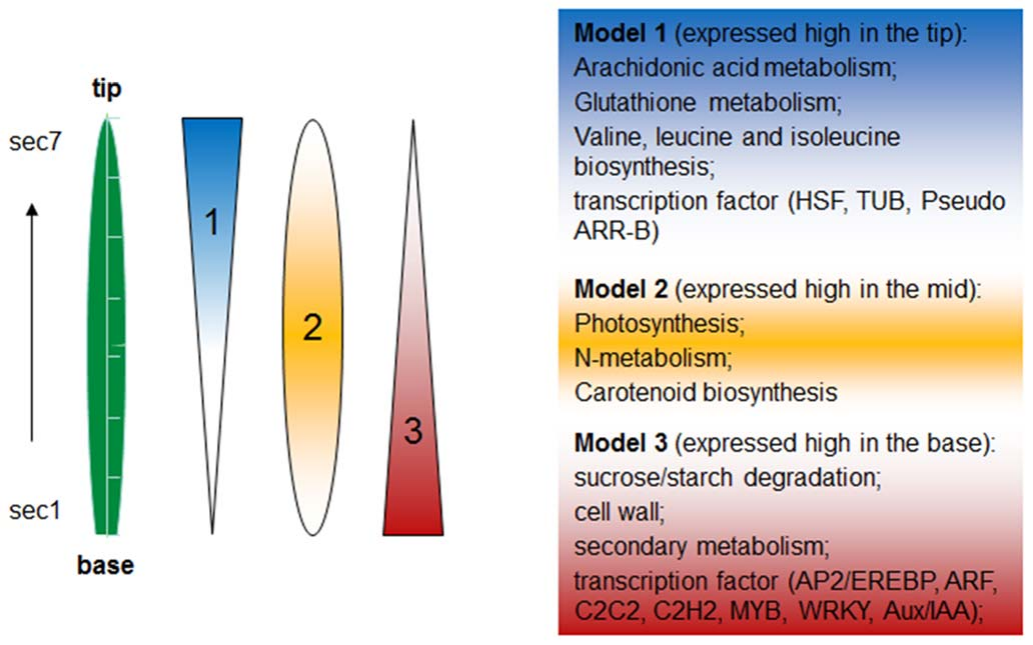
The gene expression gradient in the matured rice leaf. Schematic diagram showing 3 different models of genes differentially expressed along the leaf blade, based on the GO and KEGG enrichment analysis of the DEGs. Genes fit into model 1 are highly expressed in leaf tip, and many of them are involved in catabolism processes. Photosynthesis related genes fit to model 2, and are mainly expressed in the middle sectors. The leaf base is enriched of transcripts related to cell wall and secondary metabolism. Different families of transcription factors also showed differential gene expression patterns in the leaf base and tip.

Using a comprehensive transcriptome dataset, we have demonstrated that a nonuniform gene expression pattern exists along longitudinal axis in matured rice leaf. Gene expression analysis showed that highly expressed genes near the base are associated with primary cell wall formation, basic cellular metabolism and secondary metabolites. Many enriched pathways in the leaf base are associated with well-defined plant secondary metabolism processes, such as starch and sucrose metabolism, phenylpropanoid and flavonoid production and linoleic acid metabolism (Fig 4). In contrast, the middle sectors have the highest expression of genes involved in photosynthesis, while genes involved in catabolic processes are expressed in the leaf tip. The observed nonuniform gene expression pattern along the rice leaf suggests that the leaf cells are not homogeneous in the longitudinal axis and could have internal functional divergence. We could infer from the RNA-Seq data that the leaf base was for secondary metabolism, middle sectors were for energy production, and the tip is associated with catabolic processes. Similar observation had been made in the developing maize leaf that operates C4 photosynthesis^2^, while the current study examined the matured leaf of rice, also a monocot plant but operates C3 photosynthesis. These suggest that the segments of the monocot plant leaves, either in the matured or developing stages, are highly specialized in their biological function. However, caution should be taken, as our predication is made purely based on mRNA abundance information without the support of protein and biochemical evidence.

Previous studies have uncovered photosynthetic differences within the developing maize and rice leaves^2^,^3^. Photosynthesis related genes are highly expressed in the middle sectors of the maize leaf blade, which is the part of the leaf that contributes most exclusively to photosynthesis reactions. In our survey, we found that along the longitudinal axis of matured rice leaf, the basal and middle sectors also have higher expression levels of genes involved in photosystems I and II and carbon fixation. RuBisCO small subunit *RBCS* (LOC_Os12g17600) is the gene with the highest expression level in all sections along the mature rice leaf longitudinal axis (table S3 and S13), which reflex the general demand for RuBisCO to process C3 photosynthesis in the matured leaf. In the developing rice leaf, the *RuBisCO* expression level gradually increased from the base to tip, and became the most abundant mRNA in the middle sectors^3^. In comparison to the developing maize leaf, the key C4 enzyme coding gene *PYRUVATE HOSPHATE DIKINASE* (*PPDK*) is most abundant in the tip sections, and a similar tip to base gene expression gradient is observed for the *PHOTOSYSTE‌M II SUBUNIT R* gene^2^. These observed gene expression gradients is consistent with the photosynthetic abilities of individual leaf sections at different developmental stages. Beside these photosynthetic genes, we also found that genes involved in biotic and abiotic stresses are also differentially expressed along the matured rice leaf blade. GO enrichment analysis showed that 644 DEGs (24.74% of 2603 DEGs) are involved in responses to stresses including internal and external stimuli, biotic and abiotic stresses (Table S4). Members of stress-related transcription factor families were also found to be differentially expressed among the sections (Fig 4, Table S9). It is well known that WRKY plays important roles in disease resistance, hormone response and drought, cold and salt stresses^9^,^10^,^11^. In our study, 15 (17.44%) WRKY genes are expressed differentially along the leaf blade, and two of them showed increased expression from base to tip of leaf (Fig 3). Other TF families related to abiotic stress, such as AP2-EREBP that is known to be involved in cold-stress^12^, also showed non-uniform expression across the leaf sections (Fig. 3). Taken together, our study proves a detail insight into the dynamic nature of the rice matured leaf transcriptome, and serves a valuable resource to the research community for functional and comparative genomics studies.

## Methods

### Plant materials

The rice Nipponbare (*Oryza sativa l. japonica*) seedlings were grown in the greenhouse, with temperature at 25–28°C. About 20 days after planting, the fully opened 4^th^ leaves was cut it into seven 3-cm segments, from bottom to tip and labeled as sections 1 to 7, respectively. The tissues were immediately frozen in liquid nitrogen for total RNA extraction. Two biological replicates were collected for each section.

### RNA-Seq library preparation and sequencing

Total RNA was extracted using Qiagen Plant RNeasy kit. Illumina strand-specific RNA-Seq libraries were constructed as previously described^13^. Index libraries were pooled and sequenced.

### RNA-seq data processing

Data processing and analysis were performed as previously described^2^. Adapters were removed from raw sequence reads using FASTX-toolkit (http://hannonlab.cshl.edu/fastx_toolkit/). Sequence quality was examined using FastQC (http://www.bioinformatics.babraham.ac.uk/projects/fastqc/). Reads were mapped to the *Oryza sativa* genome (MSU Release 7.0) using Tophat^14^. Differential gene expression and alternative splicing analysis was performed by Cufflinks^15^.

### Functional categories analysis

Enrichment analyses were carried out as previously described^2^. GOslim analysis was used to determine overrepresentation of molecular function in selected groups. The number of expressed genes and DEGs connected to each GOslim category was counted. The hypergeometric test was performed on each individual GOslim category. The enrichment analysis was performed using “phyper” in R, and the *p* values from the hypergeometric test were corrected by FDR. Only GOslims with *q* < 0.05 were considered significantly enriched. AgriGO enrichment analysis was performed using http://bioinfo.cau.edu.cn/agriGO/16. KEGG enrichment analysis was performed by using KOBAS2.0^17^, and pathways with *q* < 0.05 were considered statistically significant. The OGRO annotation was retrieved from the http://qtaro.abr.affrc.go.jp/ogro database and DEGs with OGRO annotation is listed in supplementary table S12.

### Validation of RNA-Seq by qPCR

To verify RNA-seq results, real-time PCR was performed using SYBR green (Applied Biosystems) and ABI Step One Plus Realtime, quantiutative (q) PCR System (Applied Biosystems). A set of genes selected on the basis of their relatively broad distribution of expression among all sections (Table S8). Expression values by q-RT PCR were calculated by relative expression of genes to the internal control gene (*eEF-1a*)^18^.

### Accession

The data has been deposited in NCBI SRA under the accession number SRP045512 and the BAM alignment results are available at http://137.189.43.55/JBrowse/

## Author Contributions

B.L., C.X., P.L., S.Z., Z.F. and J.J.G. conceived and supervised the project. W.Y., A.Z. and N.L. performed the experiments, Y.C., W.Z. and J.X. performed the Illumina sequencing. N.L., S.Y., D.Z. and C.X. analyzed the data.

## Supplementary Material

Supplementary Information sup fig 1-4

Supplementary Information Dataset
