# Supplementary material for: Nonuniform gene expression pattern detected along the longitudinal axis in the matured rice leaf
Source: Sci Rep. 2015 Jan 26;5:8015. doi: 10.1038/srep08015 (PMC4306128; doi:10.1038/srep08015)
Supplement: Supplementary Information — sup fig 1-4 [file srep08015-s1.doc]

# Non-uniform gene expression pattern detected along the longitudinal axis in the matured rice leaf

Ning Li, Yun-Ru Chen, Zehong Ding, Pinghua Li^4^, Ying Wu, Ai Zhang, Sheng Yu, James. J. Giovannoni, Zhangjun Fei, Wei Zhang, Jenny Z. Xiang, Chunming Xu, Bao Liu and Silin Zhong


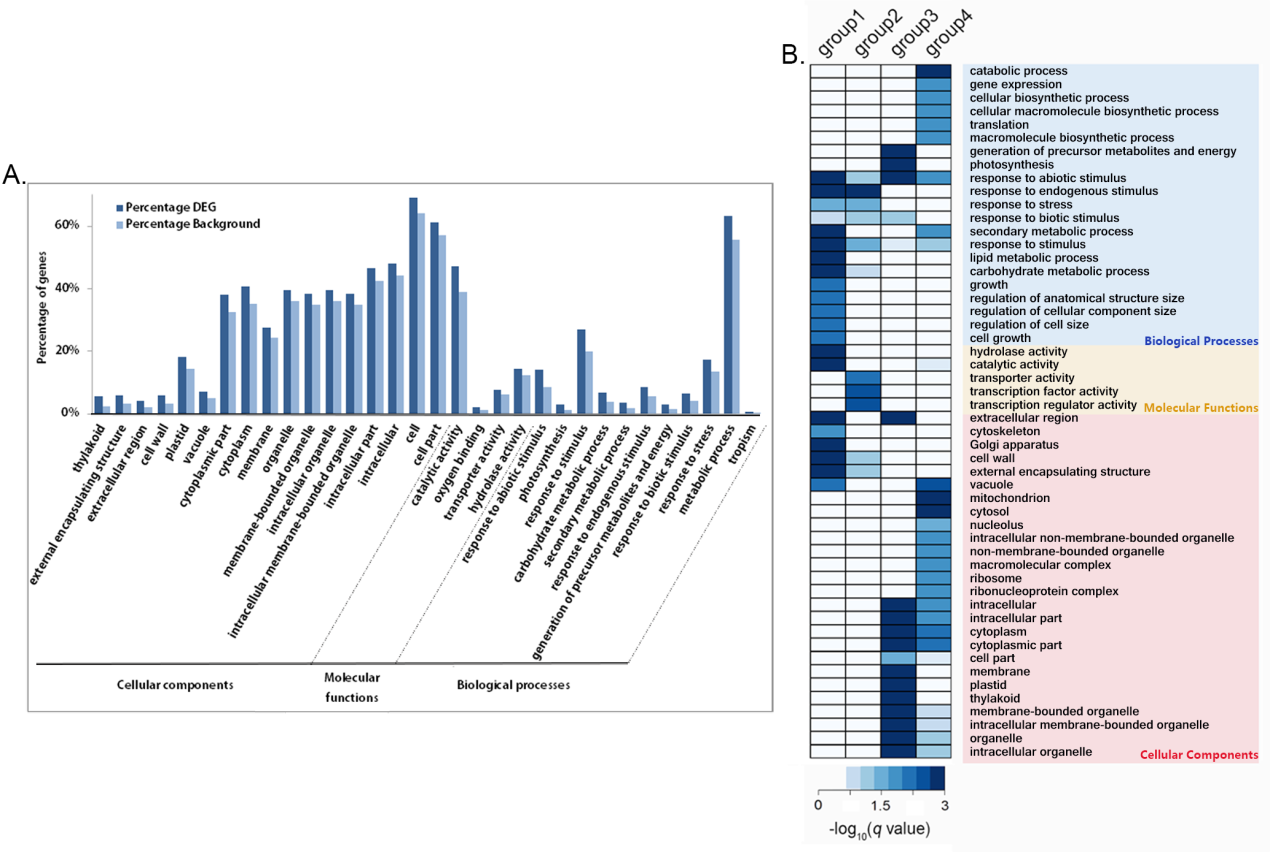


**Supplementary Figure 1 | GO enrichment of DEGs and clusters.** (A) The figure shows the significantly enriched GO iterms of DEGs. Light bars represent the percentage of expressed genes of each pathway to totally expressed GO annotated genes (approx. 21618); the dark bars represent the percentage of DEGs of each pathway to totally GO annotated DEGs (approx. 2289). (B) Heatmap of GO enrichment for the 4 categories, -log10(*q* value) of each pathway was used to draw the figure.


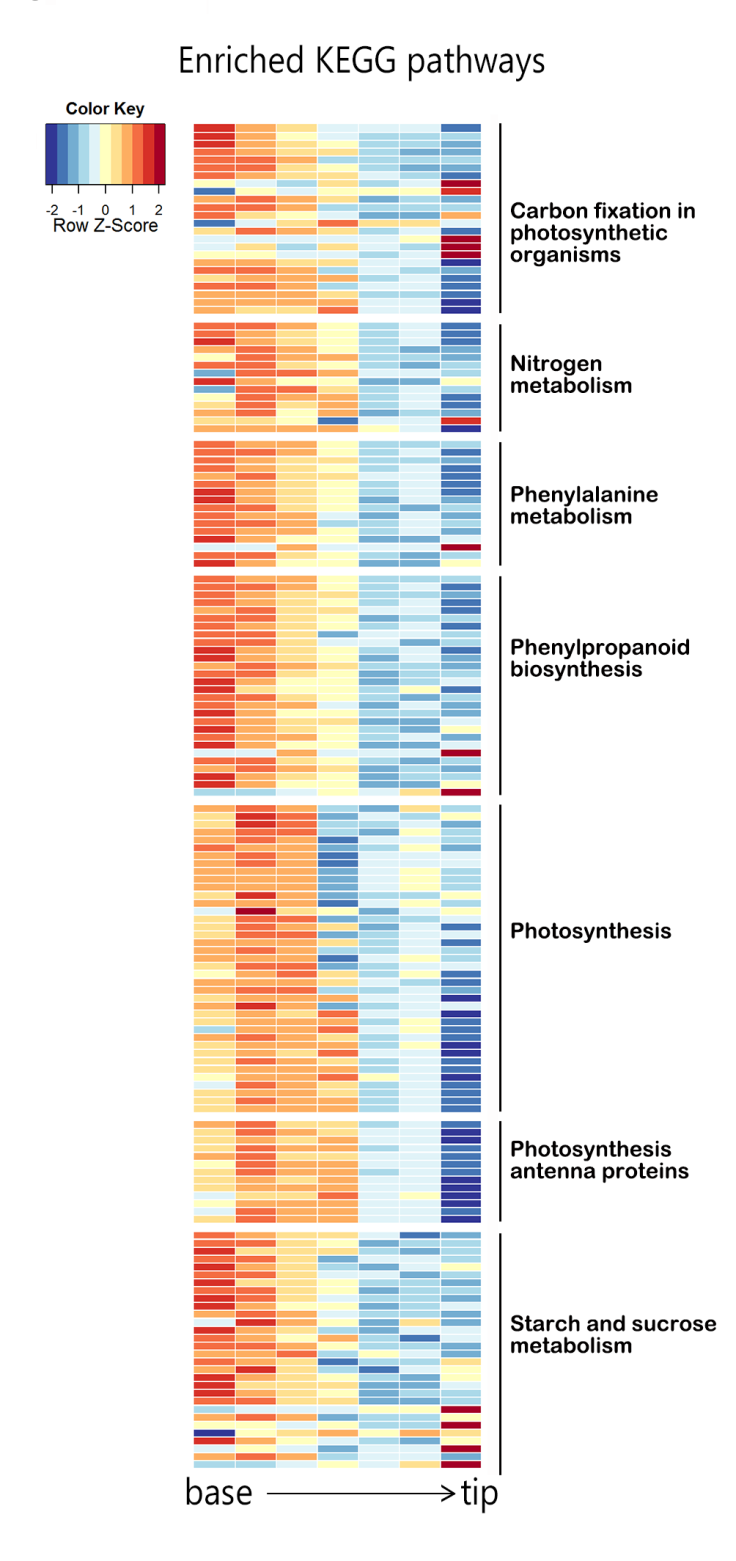


**Supplementary Figure 2 | The expression trends of differentially expressed genes of significantly enriched KEGG pathways.**


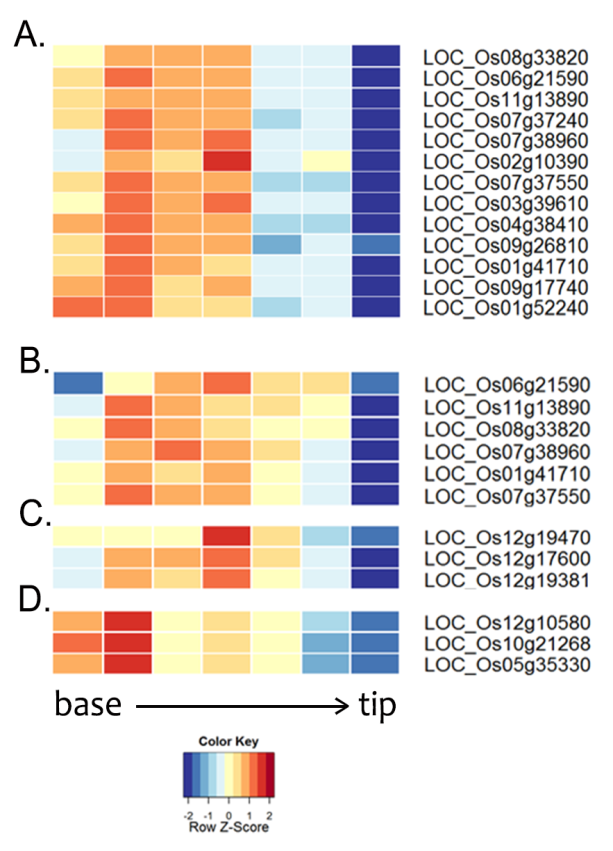


**Supplementary Figure 3 | Expression trends and validation of genes involved in photosynthesis.** (A) The expression trends of 13 differentially expressed photosynthesis antenna proteins, 6 of them were verified by q-RT PCR, and the expression trends showed in (B). (C) and (D) Represents the verification of rubisco small (RBCS) and large (RBCL) subunits genes by q-RT PCR, respectively.


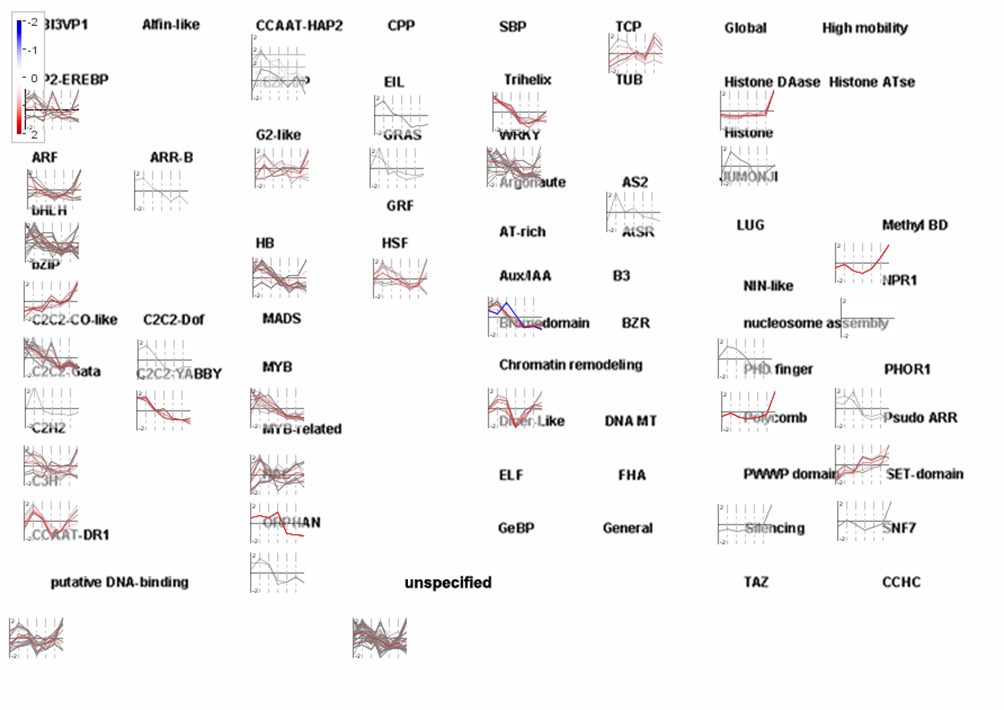


**Supplementary Figure 4 | The mapman overview of transcription factor expression in seven leaf sections.** The scaled expression levels of differentially expressed transcript factors were showed in the figure as their family attribute. The x axis refers to the seven sections (from base to tip) and y axis refers to the scaled expression level in each figure.
